# Wetting of soft superhydrophobic micropillar arrays†
†Electronic supplementary information (ESI) available. See DOI: 10.1039/c8sm01333k


**DOI:** 10.1039/c8sm01333k

**Published:** 2018-09-05

**Authors:** Periklis Papadopoulos, Bat-El Pinchasik, Martin Tress, Doris Vollmer, Michael Kappl, Hans-Jürgen Butt

**Affiliations:** a Department of Physics , University of Ioannina , Greece; b Max Planck Institute for Polymer Research , Mainz , Germany . Email: butt@mpip-mainz.mpg.de; c Department of Chemistry , University of Tennessee , Knoxville , TN , USA

## Abstract

Superhydrophobic surfaces are usually assumed to be rigid so that liquids do not deform them. Here we analyze how the relation between microstructure and wetting changes when the surface is flexible. Therefore we deposited liquid drops on arrays of flexible micropillars. We imaged the drop's surface and the bending of micropillars with confocal microscopy and analyzed the deflection of micropillars while the contact line advanced and receded. The deflection is directly proportional to the horizontal component of the capillary force acting on that particular micropillar. In the Cassie or “fakir” state, drops advance by touching down on the next top faces of micropillars, much like on rigid arrays. In contrast, on the receding side the micropillars deform. The main force hindering the slide of a drop is due to pinning at the receding side, while the force on the advancing side is negligible. In the Wenzel state, micropillars were deflected in both receding and advancing states.

## Abstract 2

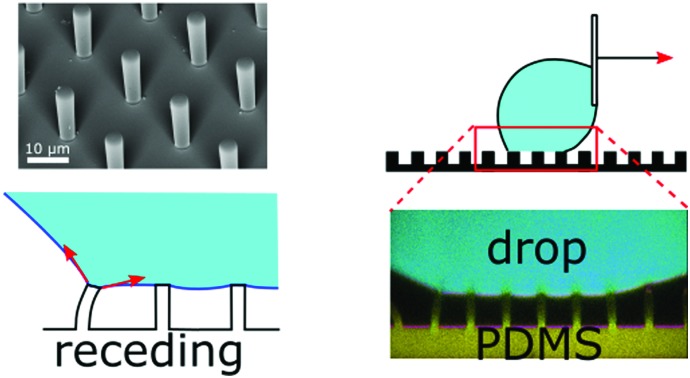
We image the bending of soft micropillars as liquid drops move on them and calculate the forces hindering drop motion.

## 1 Introduction

Most natural surfaces are flexible such as the surface of feathers and leaves or the skin of animals and humans. These surfaces are also nano- and microstructured.^1^ Flexible protrusions elastically bend and deform due to the capillary action of a liquid. Despite the omnipresence of flexible and microstructured surfaces, the understanding of the interplay between flexibility, topography, and wettability is still in its infancy. Usually the surface is assumed to be rigid so that the liquid does not deform it. It is known, however, that drops may cause deformations of a flat flexible substrate. Ridges^2^,^3^ that self-spread,^4^ wrinkles,^5^ and capillary origami^6^ are some examples. Microscopic contact angles may change due to elastocapillary effects.^7^ One of the key questions is the effect of the shape and dimensions of protrusions on the wetting properties.

To gain more insight, we investigate the interplay between elasticity and capillarity using flexible micropillar arrays. Micropillar arrays have served as model system to understand the wetting behaviour of superhydrophobic surfaces. In the superhydrophobic so-called Cassie or “fakir” state, drops partially rest on an air cushion. Lowering the surface energy or decreasing the roughness leads to a transition to the fully wetted Wenzel state. Silicone elastomers have been used for making superhydrophobic arrays as they are inherently hydrophobic and have good adhesion on glass and other substrates.^8^,^9^ Polydimethylsiloxane (PDMS) micropillars have been used as force sensors to measure drag and shear in microfluidic devices, as they show significant bending with forces in the nanonewton range.^10^–^12^ The softness of PDMS allows control of the shape and orientation by external fields, *e.g.* for smart surfaces with directional wettability, magnetic pillars,^13^ and measurement of cell adhesion forces.^14^,^15^ Currently it is unknown how these changes in shape affect wetting and superhydrophobicity. Sessile drops on flat PDMS films induced the formation of a PDMS ridge around the contact line of the drop^2^,^16^ that increases the hysteresis of the apparent contact angle.^17^

Here we measure the bending of soft PDMS micropillars that form a superhydrophobic substrate when liquid drops are placed on top. By using confocal microscopy we quantify the deflection, *i.e.* the displacement of the pillar's top face. We relate it to macroscopic wetting properties such as the apparent advancing and receding contact angles. Therefore, we moved sessile drops or increased and decreased their volume. The goal is to understand the theoretical relations between macroscopic wetting and forces acting on micropillars. These results are also relevant for hard structures with high aspect ratio that are usually considered stiff, but may bend due to capillary forces or when drops are deposited or evaporate on them.

## 2 Experimental

### 2.1 PDMS micropillars

**Fig. 1:**
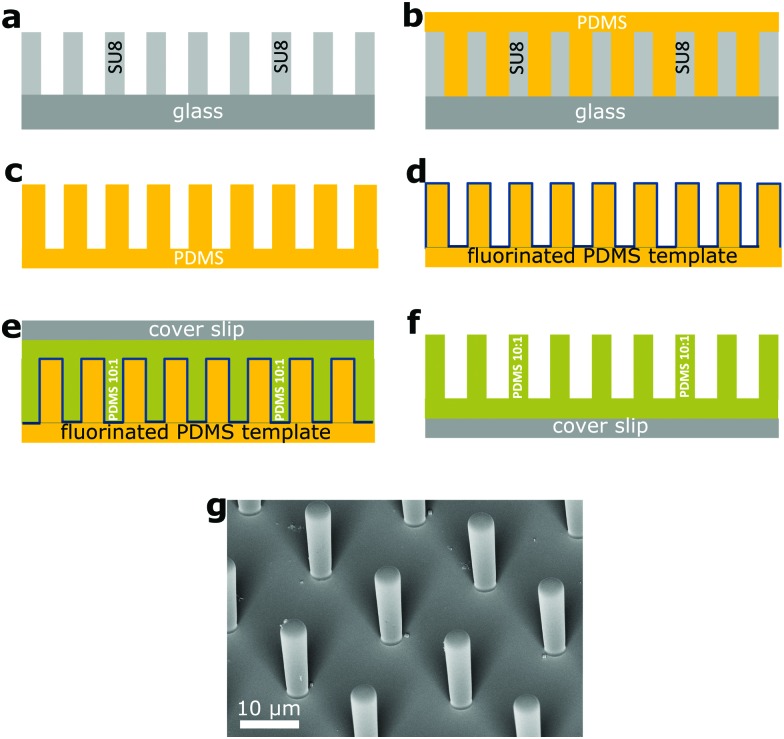
Fabrication of PDMS micropillar arrays. (a) A hard template of cylindrical micropillars on 1 mm thick glass (gray) was made with photolithography with the SU-8 photoresist (Microchem).^20^ (b) From this template we made a thick and flexible PDMS template with cylindrical holes (c). (d) The surface of the PDMS template was fluorinated (the blue line) with 1*H*,1*H*,2*H*,2*H*-perfluorooctyltrichlorosilane by chemical vapor deposition to reduce the surface energy and render it non-sticking. (e) We poured the PDMS monomer/crosslinker mixture (Sylgard 184, a mixing ratio of 10 : 1) with perylene diimide dye at a concentration of 0.2 mg ml^–1^ on the PDMS template (green). After degassing we pressed the 170 μm cover slip on it, to remove excess liquid PDMS. (f) After curing at 60 °C for 12 h, the soft PDMS template was peeled off, leaving the PDMS pillars on the cover slip. (g) Scanning electron microscope image of a square array of cylindrical PDMS micropillars (*a* = 20.0 μm, *d* = 4.0 μm, *h* = 14.0 μm).

We fabricated square arrays of PDMS micropillars with circular cross-sections (Table 1) directly on 170 μm precision cover slips (Carl Roth) by two-step soft lithography (Fig. 1). In some samples with high aspect ratio a large number of micropillars collapsed^18^ and they recovered using ultrasound.^19^ The final material had a Young's modulus of *E* = 1.0 MPa, as measured with a ProLine Z005 rheometer (Zwick-Roell, Germany) for a 10 × 10 mm^2^ sample.

**Table 1:** Pillar height *h*, diameter *d*, center-to-center distance *a*, and micropillar spring constant *k* for each sample

Sample no.	*h* (μm)	*d* (μm)	*a* (μm)	*h*/*d*	*k* (N m^–1^)
**1**	17.5 ± 0.3	4.5 ± 0.3	20 ± 0.5	3.9	0.012 ± 0.003
**2**	14.0	4.5	20	3.1	0.022 ± 0.006
**3**	10.0	5.0	20	2.0	0.09 ± 0.02
**4**	10.0	4.0	20	2.5	0.038 ± 0.012
**5**	17.0	10.0	40	1.7	0.30 ± 0.04

**Fig. 2:**
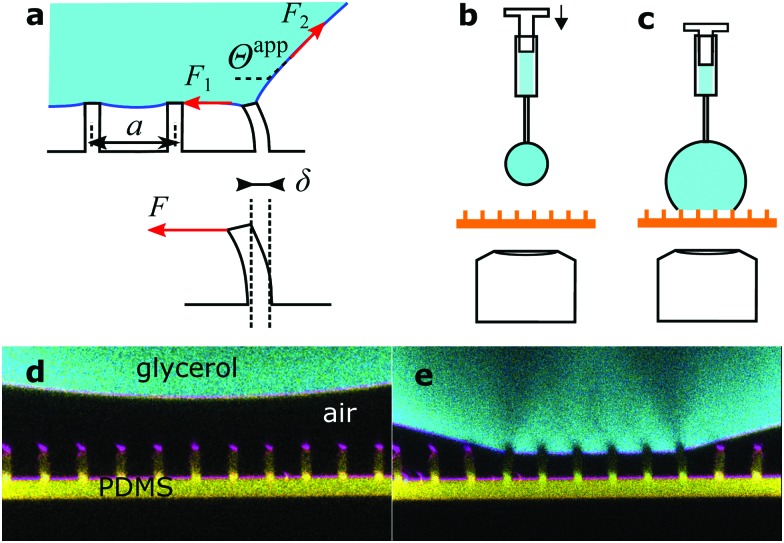
Drops of glycerol on arrays of PDMS micropillars. (a) Forces acting at the macroscopic contact line. Microscopically forces are exerted by the liquid surface (red arrows) and are determined by the apparent contact angle, *Θ*^app^ (eqn (5)). The force corresponding to the center-to-center distance *a* is actually applied to a single pillar that is deflected by *δ*, as there is no other contact with the solid. (b) A pendant drop is hanging from the needle of a syringe and approaches the micropillars. (c) As the drop size increases, the bottom of the drop touches the top faces of the micropillars. Advancing and receding apparent contact angles are measured by increasing or decreasing the volume of the drop with the syringe, respectively. (d) Confocal image of a pendant glycerol and (e) sessile drop on sample **1**. Glycerol in cyan, PDMS in yellow, and reflected light in magenta. The pillar center-to-center distance is 20 μm everywhere.

Pillar deflection is directly related to capillary forces. In the Cassie state, capillary forces are exerted at the edge of the top face of wetted micropillars. Assuming a horizontal point force load *F* at the free end of a cylindrical micropillar with height *h* and diameter *d* (Fig. 2a), the horizontal deflection *δ* is given by^21^,^22^
1
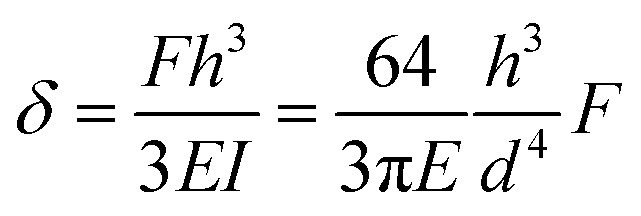
where *I* = π*d*^4^/64 is the area moment of inertia. The expected shape of the micropillar is a curve described by the following equation for the displacement of the center *x* as a function of height *z*:2
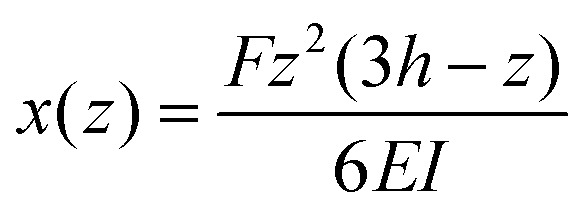
We can define the spring constant (Table 1) of the micropillars as3
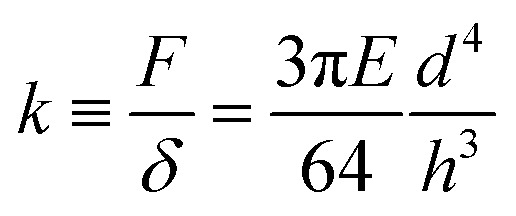
These relations are strictly valid only for small deflections (*δ* ≪ *h*) and long pillars (*d* ≪ *h*). We examine below how well they can describe the present system.

### 2.2 Liquids

To prevent the change of drop volume in the course of measurements we used a glycerol/water mixture, 80 : 20 vol/vol, which is at equilibrium with ambient air at 50% RH. Its surface tension was 0.065 N m^–1^. Glycerol has roughly the same refractive index (*n*_L_ = 1.45) as PDMS (*n*_PDMS_ = 1.43)^23^ and high surface tension (*γ*_L_ = 64 mN m^–1^),^24^ does not swell PDMS,^25^ and does not evaporate. For confocal measurements drops were labeled fluorescently with the Alexa488 dye (Invitrogen) at a concentration of 0.2 mg ml^–1^. For simplicity, this mixture is simply referred to as “glycerol” in the text. For an experiment, a drop of 5 μL was placed by a fixed syringe (Hamilton) onto a micropillar array (Fig. 2b). The position of the array was controlled with a motorized *xy*-stage. To deposit glycerol onto micropillar arrays a pendant drop, hanging from the needle, increased in size, until the bottom of the drop touched the top of a small number of pillars (Fig. 2). All drops deposited this way were in the Cassie state.

### 2.3 Confocal microscopy

For confocal imaging we used a custom-made inverted microscope, with excitation lasers at 473 and 532 nm, and three objectives: 40×/0.85 dry, 40×/1.30 water immersion and 63×/1.30 glycerol immersion (Olympus). Both reflectance and fluorescence images were acquired simultaneously, either as 2D *xz*-slices or full 3D stacks. In air, images are obscured by reflections from the top faces of the micropillars and fluorescent intensity decreases with height. To suppress reflections we made another set of measurements with glycerol drops immersed in a fluorinated oil, Fluorinert FC-40 (3M). This oil is immiscible with glycerol, has low surface tension, and does not swell PDMS.^25^ Even though it has a slightly lower refractive index *n*_FC40_ = 1.29 than PDMS and glycerol, reflections were suppressed. Images improved significantly so that the shape of the whole pillar could be observed. In confocal microscopy axial distances are measured correctly only when the light beam passes through a medium with the same refractive index as the objective is designed for. Thus, for correct measurement of the apparent contact angle *Θ*^app^ of glycerol drops in air we used a dry objective when *Θ*^app^ > 90° and glycerol immersion when *Θ*^app^ < 90°. For glycerol drops in FC-40 we used instead the water immersion objective when *Θ*^app^ > 90°, as the refractive index of FC-40 was close to that of water (*n*_H_2_O_ = 1.33).

As confocal microscopy resolves the three-dimensional shape of the pillars, we distinguish between the material's contact angle *θ* measured at a length scale on the order of the resolution of the microscope (<1 μm), and the apparent contact angle *Θ* at length scales larger than the size of the pillars. The former is measured on a flat PDMS surface, prepared in the same way as the pillars. The latter is defined as the angle between the horizontal and a fitted straight line on the drop–air or drop–FC-40 interface (Fig. 2a). Both microscopic and apparent contact angles exhibit hysteresis. The advancing and receding material's contact angles of sessile glycerol drops on PDMS surrounded by air were *θ*_adv_ = 111 ± 3° and *θ*_rec_ = 61 ± 3°, respectively, compared to *θ*_adv_ = 122 ± 3° and *θ*_rec_ = 90 ± 3° for glycerol drops immersed in FC-40. The stability of the Cassie state in FC-40 was higher because the material's contact angles were higher.

## 3 Results and discussion

**Fig. 3:**
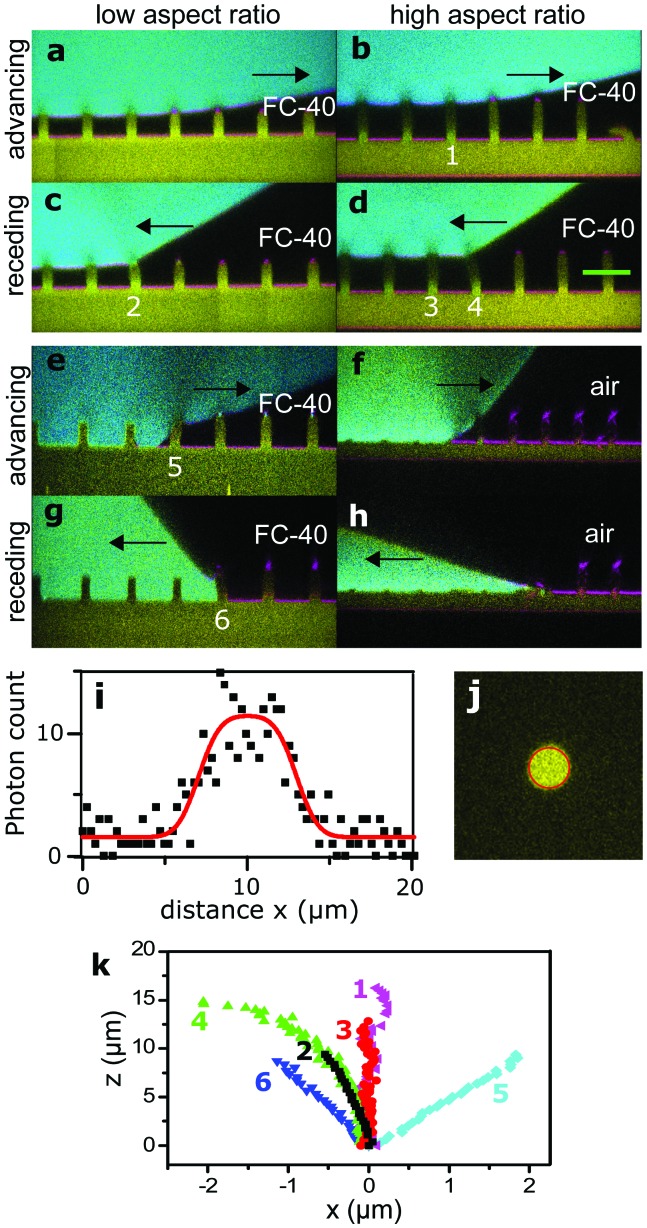
Advancing and receding states of drops. Arrows show the direction of motion of the wetting front. Glycerol in cyan, PDMS in yellow, and reflected light in magenta. Black is either air or FC-40. (a) Advancing Cassie state on sample **3** in FC-40, see Table 1. (b) Advancing Cassie state on sample **1** in FC-40. (c and d) Respective receding states. (e) Advancing Wenzel state on sample **4** in FC-40. (f) Advancing Wenzel state on sample **2** in air. The advancing front caused a permanent collapse of the pillars. (g and h) Respective receding Wenzel states. (i) Intensity profile along the green line in (d), fitted as described in the text (red line). (j) Horizontal cross-section of a micropillar, fitted with a 2D function (red circle, see text). (k) Shape of pillars numbered in (b–f).

We placed drops on arrays of micropillars and we analyzed the bending of micropillars under the drop, at the macroscopic contact line and outside the drop, while the drop advanced or receded. As-deposited drops were always in the Cassie state, but it was also possible to trigger the Cassie-to-Wenzel transition by letting the drops fall from a height of about 2 cm. First, we acquired series of *xz* images with the confocal microscope on the advancing and receding sides of drops, on micropillars with different aspect ratios in either wetting state (Fig. 3).

The shape of a PDMS micropillar was analyzed by extracting the position of the center of horizontal micropillar cross-sections at different heights. As an example, we plot the intensity profile along the green line of Fig. 3d in Fig. 3i. The intensity profile *I*(*x*) was fitted with the convolution of a rectangular function with a Gaussian point-spread function (red line):4
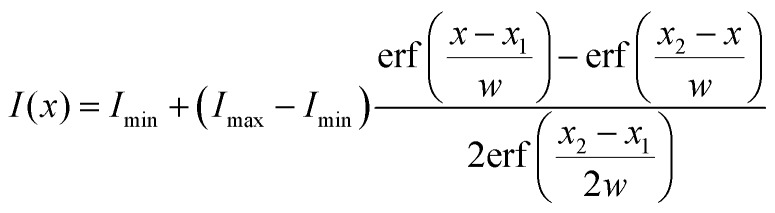
where *I*_min_ and *I*_max_ are the minimum and maximum intensity of the profile, *x*_1_ and *x*_2_ the left and right boundaries of the pillar, respectively, and *w* the FWHM of the point-spread-function. The position of the center is then calculated as *x*_0_ = (*x*_1_ + *x*_2_)/2. The typical uncertainty of *x*_0_ is about 200 nm. This fit was repeated at different heights to get the complete pillar shape *x*_0_(*z*), Fig. 3i, and the displacement *δ* = *x*_0_(*z*_max_). For better accuracy, we acquired complete 3D stacks of confocal images under quasi-static conditions. In this case, we fitted complete *xy*-slices of micropillars at different heights with the convolution of a circle with a two-dimensional Gaussian point-spread function (Fig. 3j, red circle). Here the displacement is a 2D vector. Typical uncertainties are on the order of 50 nm, as a much larger number of pixels is fitted compared to the one-dimensional profile in Fig. 3i.

On the advancing side of a drop, pillars at the macroscopic contact line showed no measurable deflection and remained vertical (Fig. 3k, pillar 1). Also the inner pillars under the drop were not bent (Fig. 3k, pillar 3). In the latter case, the standard deviation of the center position is *σ*_*x*_ = 50 nm, which gives the force resolution as 0.7 nN for sample **1**, assuming that eqn (3) is valid. Thus, we conclude that no net horizontal force acts on those pillars and that the advancing side does not influence the sliding of the drop. This agrees with previous results of rigid superhydrophobic micropillar arrays, in which drops advancing in the Cassie state advance by touching down on the top of the next row of pillars, practically at 180°.^26^

**Fig. 4:**
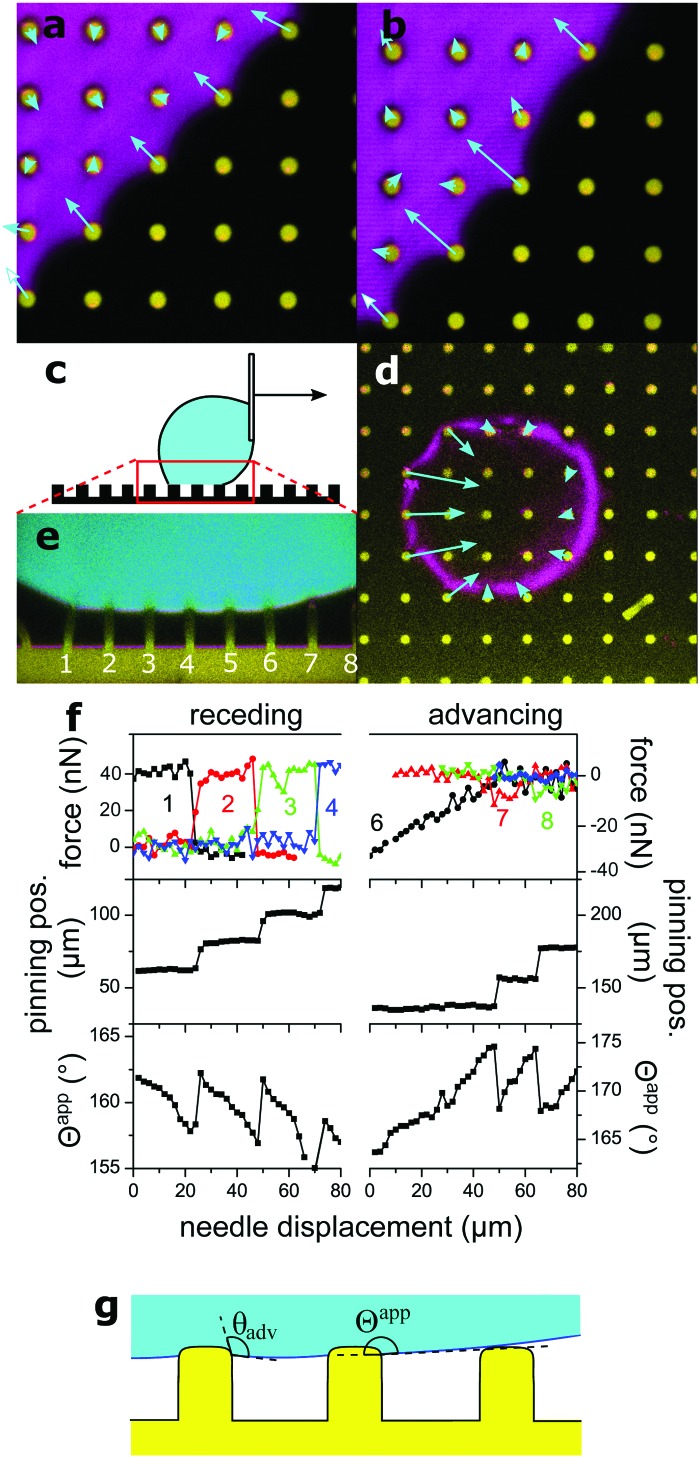
Micropillar deflection at the contact line of a glycerol drop moving to the right. (a) Sample **3** in the receding Cassie state in FC-40, *xy* cross-section, fluorescence of pillars in yellow, projection of reflection from a height range ±1 μm from the top of the pillars, in magenta. Vectors show the deflection at the top of each pillar, magnified by 20. (b) Same as (a), with a dislocation in the macroscopic contact line. (c) Schematic of the moving drop. (d) Sample **1** in FC-40, *xy* cross-section. Vectors show deflections of pillars at the macroscopic contact line, magnified by 10. (e) *xz* cross-section from the same experiment, needle displaced by 20 μm. Same colors as Fig. 3. Deflection of numbered pillars was analyzed as a function of needle displacement. (f) Capillary force, position of the macroscopic contact line, and apparent contact angle *Θ*^app^ as a function of needle displacement. The drop was initially symmetric between the advancing and receding states. Force curves correspond to the numbered pillars in (e). Force was calculated from the deflection, assuming a spring constant of 0.012 N m^–1^ (Table 1). Positive forces are to the right. The lower value of *Θ*^app^ at a displacement of 70 μm on the receding side is due to a small movement of the center of the drop along the *y*-axis, normal to the image. (g) The liquid wets the vertical walls of micropillars until the material's advancing contact angle *θ*_adv_ is reached. An advancing drop touches the top face of the next pillar before the drop–air interface becomes horizontal, thus the apparent contact angle *Θ*^app^ is slightly lower than 180°.

To get an impression about the magnitude and direction of capillary forces at different pillars, we assume at this point that the calculated spring constant (eqn (3), Table 1) gives the correct force to displacement ratio. In the Cassie state, the deflection-to-height ratio *δ*/*h* never exceeded 0.2. Therefore, we expect a linear dependence between deflection and capillary force, according to eqn (1). Thus, deflection maps are essentially force maps (Fig. 4a and b). When the macroscopic contact line followed a long straight continuous path along a row of pillars, forces were equal for each pillar at the contact line (Fig. 4a). During receding, step-by-step depinning (Movie S1, ESI†) created a contact line that jumps from one row to the next. Deflection is higher at discontinuities and points where the macroscopic contact line changes orientation (Fig. 4b and d), when for example the contact line changes from one row to the next row of micropillars.

To follow capillary forces during advancing and receding, we moved the needle which held the drop horizontally at constant speed with a motorized stage (Fig. 4c) while imaging. In Fig. 4d and e, for example, the drop moves to the right; the left side of the drop is receding and the right is advancing. Capillary forces are high on the receding side, but practically zero on the advancing (Fig. 4d), in agreement with previous theoretical work.^27^ As the drop moved, stepwise depinning occurred on the receding side (Fig. 4f). After each jump, the pillar at the new contact line reached almost immediately the maximum deflection.

The apparent contact angle is measured from the *xz* cross-section at a height of 5 μm above the pillars, to exclude variations due to the details of the microscopic contact line.^28^ On the advancing side, jumping of the contact line occurs independently of the receding side and deflections are nearly zero. Only a small and short-lived snap-in deflection just after a jump was observed, due to the fact that *Θ*^app^ is slightly less than 180°. The apparent advancing contact angle was close to 175° instead of being exactly equal to 180°, because the edges of PDMS are rounded and the liquid wets a small part of the vertical walls (Fig. 1g and 4g).

So far we assumed that the spring constant of eqn (3) can be used to calculate force from deflection in the Cassie state. We now check, first, that micropillar deflection is proportional to the force acting on the top face and, second, if eqn (3) gives the correct spring constant, despite the fact that micropillars are not infinitely thin beams. Therefore we calculate the capillary force *F* on single micropillars independently from pillar properties, from the shape of the liquid meniscus (Fig. 2a). For a contact line along a row of micropillars as in Fig. 4a, the mean horizontal force per unit length in the Cassie state is *f* = *γ*(1 + cos *Θ*^app^). It is determined by *Θ*^app^ and the surface tension *γ*,^27^ which is here the glycerol–air (*γ* = 65 mN m^–1^) or glycerol–FC-40 interfacial tension (*γ* = 35 mN m^–1^). The first term, *γ*, corresponds to the horizontal liquid surface to the next wetted micropillar (*F*_1_, Fig. 2a). The second term, *γ* cos *Θ*^app^, is the horizontal component of the capillary force at the outer drop interface (*F*_2_, Fig. 2a). Thus, if the contact line is pinned along the main axis of the array without any discontinuities, the horizontal force acting on a single micropillar *F* is proportional to the center-to-center distance *a*:

5 *F* = *aγ*(1 + cos *Θ*^app^)

**Fig. 5:**
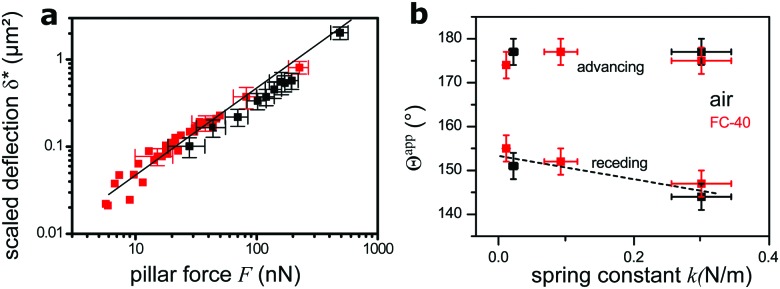
Dependence of deflection on force. (a) Scaled deflection δ* (eqn (6)) as a function of force on a single pillar, as calculated from the apparent contact angle (eqn (5)). Measurements from both static (Fig. 3) and moving substrate experiments (Fig. 4) are summarized. Drops were always in the Cassie state, either in air (black) or in FC-40 (red). The straight line, with slope of 1, is the theoretical deflection of bending beams with *E* = 1.5 MPa. Some error bars were omitted for clarity. (b) Dependence of the apparent contact angle *Θ*^app^ of Cassie drops on the micropillar spring constant. Glycerol drops in air and FC-40 are shown in black and red, respectively. The dashed line is a guide for the eye.

As the hysteresis of *Θ*^app^ was about 30° for all samples (Fig. 5b) it was possible to vary *F* within a broad range, by *e.g.* starting with a drop forming the receding *Θ*apprec and slowly increasing *Θ*^app^ by moving the drop until *Θ*^app^ = *Θ*appadv and the contact line jumped to the next row of pillars. The analysis of movies of *xz*-confocal images (Movie S2, ESI†) gives the deflection and *Θ*^app^. Velocities were on the order of 1 μm s^–1^, so measurements can be considered quasi-static. To compare deflections of different micropillars one needs to consider the pillar dimensions. Thus, we define the scaled deflection6
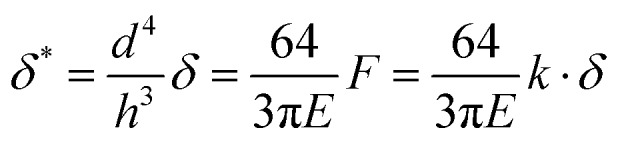



We plot the scaled deflection against the horizontal capillary force *F* (eqn (5)) in Fig. 5a. All experiments show the same linear dependence, despite varying pillar dimensions and interfacial tension. The fit gives a Young's modulus of *E* = 1.5 ± 0.3 MPa. This result is nearly equal to the value of 1.0 MPa for the same material measured by the rheometer. The slightly higher value implies that deflections are lower than the prediction of eqn (1), because the aspect ratio of pillars is not high enough, as it was assumed.

Finally, we compare the apparent contact angles on different samples. As all samples were made of the same material, the spring constant of micropillars is controlled by their aspect ratio. For drops in the Cassie state, advancing contact angles do not vary with the force constant, as the micropillars are not significantly bent at the advancing side. At the receding side, pillar deflection increases with a decreasing spring constant, causing an increase of the apparent receding contact angle compared to stiffer pillars (Fig. 5b).

## 4 Conclusions

In conclusion, drops advance on flexible superhydrophobic micropillar arrays by touching down on the next top faces of micropillars, much like on rigid arrays. Only small horizontal forces are exerted by the advancing drop on the micropillars. In contrast, on the receding side the micropillars deform, going in hand with higher apparent receding contact angles. The resulting force acting in a horizontal direction hinders a drop to slide. Micropillar arrays can be used to measure the horizontal force around the macroscopic contact line of a sessile drop in the Cassie state. The combination of soft substrates with confocal microscopy and drop adhesion force measurements may open up new opportunities to control wetting and tune the stability of the Cassie state.

## Conflicts of interest

There are no conflicts to declare.

## Supplementary Material

Supplementary movie

Supplementary movie
